# Effects of the timing of the initiation of dietary intake on pediatric type 1 diabetes for diabetic ketoacidosis

**DOI:** 10.1186/s12887-022-03243-z

**Published:** 2022-04-13

**Authors:** Xuewen Yuan, Jieguo Wang, Xiaofeng Chen, Wu Yan, Qing Niu, Ning Tang, Ming Zhi Zhang, Wei Gu, Xu Wang

**Affiliations:** 1 Department of Endocrinology, Children’s Hospital of Nanjing Medical University, 72 Guangzhou Rd, Nanjing, 210008 China; 2 Department of Emergency, Pediatric intensive care unit, Children’s Hospital of Nanjing Medical University, Nanjing, 210008 China; 3 Department of Orthopedics, Children’s Hospital of Nanjing Medical University, Nanjing, 210008 China; 4 Department of Children Health Care, Children’s Hospital of Nanjing Medical University, Nanjing, 210008 China; 5 Children’s Hospital of Fudan University, Shanghai, 201102 China; 6 School of Public Health, Nanjing Medical University, Nanjing, 211166 China

**Keywords:** Type 1 diabetes, Diabetic ketoacidosis, The timing of the initiation of dietary intake, China, Children

## Abstract

**Background:**

Precision treatment of pediatric diabetic ketoacidosis (DKA) has been the focus of research for decades. Whether the timing of the initiation of dietary intake contributes to DKA correction is ignored.

**Methods:**

We conducted a retrospective study to investigate the effects of the timing of the initiation of dietary intake on DKA correction in Children’s Hospital of Nanjing Medical University, a tertiary children’s hospital, from June 2017 to December 2020. Individual basic characteristic and clinical information of all DKA cases (*n* = 183) were collected. Multiple linear regression, logistic regression model and random forest (RF) model were used to assess the effect of the timing of the initiation of dietary intake on DKA correction.

**Results:**

The mean age of the children diagnosed with DKA was 6.95 (SD 3.82) years. The median DKA correction time and the timing of the initiation of dietary intake was 41.72 h and 3.13 h, respectively. There were 62.3% (*n* = 114) patients corrected DKA at the end of the 48-h rehydration therapy. For each hour delay in starting dietary intake, child’s DKA correction was prolonged by 0.5 (95% CI 1.05, 1.11, *P* < 0.001) hours and the adjusted odds of DKA over 48 h was increased by 8% (OR = 1.08, 95% CI: 1.05, 1.11, *P* < 0.001) after adjustment for potential confounders. The RF model based on the timing of the initiation of dietary intake and child’s weight and systolic pressure achieved the highest AUC of 0.789.

**Conclusion:**

Pediatricians should pay attention to the effect of the timing of the initiation of dietary intake, a controllable factor, on DKA correction.

**Supplementary Information:**

The online version contains supplementary material available at 10.1186/s12887-022-03243-z.

## Introduction

In the past a few decades, disease spectrum in children has changed significantly. Infective diseases, parasitic diseases, malnutrition anemia are no longer major threats to children’s health, while the incidence of endocrine and metabolic diseases continues to rise, with pediatric type 1 diabetes mellitus (TID) being of greater concern. Worldwide estimates of numbers of pediatric TID continue to increase [1]. In China, the incidence of TID was 1.93 per 100,000 person years under the age of 14 during 2010-2013 [2]. Diabetic ketoacidosis (DKA) is a common acute and severe complication of pediatric TID, and is associated with mortality. The incidence of DKA in newly diagnosed children with TID is related to region, socioeconomic status and age of onset, ranging from 15 to 70% [3].

DKA poses a great threat to children’s life in the acute phase, and also has an impact on children’s long-term health [4]. The proportion of children who have significant brain injuries during DKA are relative rare, with 0.5-0.9%. Importantly, some children who had no significant decline in neurological function during DKA therapy showed neurological changes after recovery, including deficits in memory, attention, IQ and brain microstructure [4–9]. Therefore, in order to standardize the therapy of DKA and reduce the mortality rate and the incidence of serious adverse events in children, therapy protocols of DKA were formulated according to the retrospective studies, including emergency evaluation based on clinical information, resuscitation in severe cases, appropriate administration of intravenous rehydration, maintaining electrolyte balance, intravenous insulin therapy, close monitoring of clinical state and so on [10].

To date, many prospective studies were designed to explore the dosage or rates of intravenous rehydration [4, 11] or different insulin treatments on pediatric DKA [12, 13]. However, in clinical practice, we found that the time when children started dietary intake may also be valuable for the correction of DKA, but the real-world studies on this topic are scarce. Hence, we conducted a retrospective study to discover the effects of the timing of the initiation of dietary intake on DKA correction.

## Methods

### Population

The retrospective study was approved by the Medical Ethics Committee of Children’s Hospital of Nanjing Medical University (202101014-1). Signed informed consents were obtained from parents or legal guardian for study participation. All methods were carried out in accordance with relevant guidelines and regulations under ethics approval and consent to participate. All DKA cases (*n* = 183) were collected from Children’s Hospital of Nanjing Medical University, a tertiary children’s hospital, from June 2017 to December 2020. Eligibility criteria were as follow: 1) Blood glucose level ≥ 11.1 mmol/L; 2) Arterial pH < 7.3, and/or arterial HCO_3_^−^ < 15 mmol/L; 3) Ketoemia and/or ketouria; 4) DKA treatment followed established procedures for mild, moderate, and severe DKA [14]. In addition, all cases were new-onsetT1D with ketoacidosis. Serum diabetes-related antibodies were tested in all children with diabetes. The included cases had to be positive for at least one of the three islet autoantibodies (glutamate decarboxylase antibody, insulin autoantibody, and islet cell antibody). Type 2 diabetes, non-autoimmune diabetes and cases that developed cerebral edema during correction of DKA was excluded. We did not intervene in the timing of the initiation of dietary intake subjectively. Children began to intake according to their own conditions.

### Data collection

Individual basic characteristic (child’s age, gender, height, weight, feeding method, maternal parity, gestational age, delivery method and family diabetes) were acquired at the time of admission. During the time of hospitalization, we recorded the timing of the initiation of dietary intake and DKA correction time, as well as the clinical information of child’s blood pressure, degree of dehydration, HbA1c (Bole Company, American), insulin and C-peptide (Roche GmbH, Germany) on the first day of hospitalization. Arterial PH (Radiometer, Denmark), arterial HCO_3_^−^ (Radiometer, Denmark), blood glucose (Bayer Healthcare LLC, Germany) and blood ketone (Abbott Diabetes Care Ltd., American) were collected at multiple time points within 48 h. All medical data were reviewed at least twice to ensure consistency. The time children began to intake food containing calories were counted as the timing of the initiation of dietary intake, and the specific time was recorded and checked by children’s nurses and endocrinologists. DKA was considered corrected in the presence of: arterial pH > 7.3, arterial HCO_3_^−^ ≥ 15 mmol/L, as well as no ketoemia and ketouria. DKA status was assessed at 48 h as a cut-off point at the end of 48-h rehydration therapy [15].

### Statistical analysis

The individual basic features of 183 DKA cases were presented as Mean ± SD for continuous variables, and frequency and percentages for categorical variables. T-test were used for continuous variables and chi-square test for categorical variables.

Multiple linear regression model was used to assess the association between the timing of the initiation of dietary intake (continuous variables, independent variables) and DKA correction time (continuous variables, dependent variables). Logistic regression model was used to estimate the associations between the timing of the initiation of dietary intake (continuous variables, independent variables) and odds of corrected DKA over 48 h (categorical variables, independent variables). Model 1 was unadjusted; model 2 was controlled for potential confounders, including child’s age, gender, weight, height and degree of dehydration. Inclusion of confounders depends on the simple linear regression (standard: *P* < 0.1) and the prior knowledge from the scientific articles. Further, the independent effects of the timing of the initiation of dietary intake were analyzed, as well as the timing of the initiation of dietary intake combined with other factors on DKA correction using a random forest (RF) model. The data set adopted 10-fold cross validation. The RF model was implemented using R. version 3.2.2 (R Core Team 2014) using the “random Forest” package. The graphical Relative Operating Characteristic (ROC) curve is produced, and the area under the ROC curve (AUC) is a performance index to measure the effectiveness of the model.

## Results

In 183 cases, individual basic features were described in Table 1. The mean age of children diagnosed with DKA was 6.95 (SD 3.82) years. There were 62.3% (*n* = 114) patients corrected DKA at the end of the 48-h rehydration therapy, while 37.7% (*n* = 69) corrected DKA for more than 48 h. Children in the group with corrected DKA for more than 48 h were heavier on admission than within 48 h. (Mean ± SD: 24.6 ± 11.8 vs 20.9 ± 8.7, *P* = 0.025). Also, in the group with corrected DKA over 48 h, children were admitted with higher systolic blood pressure than within 48 h. (Mean ± SD: 116.0 ± 13.0 vs 111.0 ± 11.0, *P* = 0.001). No significant differences were observed between corrected DKA over 48 h and within 48 h in child’s age, gender, height, feeding method, maternal parity, gestational age, delivery method, family diabetes, diastolic pressure, HbA1c, insulin and C-peptide. In addition, the degree of dehydration did not differ between groups with different DKA correction time.

**Table 1:** Individual basic characteristic of 183 pediatric type 1 diabetes for diabetic ketoacidosis

		DKA ≤ 48 h (*n* = 114)	DKA > 48 h (*n* = 69)	*P*
Child’s age (years)		6.52 ± 3.65	7.51 ± 4.05	0.090
Child’s height (cm)		117.7 ± 23.4	125.2 ± 26.6	0.070
Child’s weight (kg)		20.9 ± 8.7	24.6 ± 11.8	**0.025**
Child’s gender	Boy	57 (50)	29 (42)	0.295
	Girl	57 (50)	40 (58)	
Child’s feeding method	Breast milk	65 (57)	43 (62.3)	0.700
	Artificial feeding	17 (14.9)	11 (15.9)	
	Mixed feeding	32 (28.1)	15 (21.7)	
Maternal parity	1	71 (62.3)	46 (66.7)	0.49
	2	41 (36)	23 (33.3)	
	≥3	2 (1.8)	0 (0)	
Maternal gestational age	Full-term	110 (96.5)	62 (89.9)	0.186
	Preterm	3 (2.6)	5 (7.2)	
	Retard	1 (0.9)	2 (2.9)	
Maternal delivery method	Natural delivery	56 (49.1)	26 (37.7)	0.171
	Cesarean section	58 (50.9)	42 (62.3)	
Family diabetes history	No	74 (64.9)	50 (72.5)	0.289
	Yes	40 (35.1)	19 (27.5)	
Child’s systolic pressure (mmHg)		111.0 ± 11.0	116.0 ± 13.0	**0.011**
Child’s diastolic pressure (mmHg)		70.0 ± 9.0	73.0 ± 12.0	0.128
HbA1c at admission (%)		12.50 ± 1.71	12.3 ± 1.70	0.504
Insulin at admission (mU/L)		13.20 ± 34.19	15.67 ± 52.80	0.711
C-peptide at admission (nmol/L)		0.11 ± 0.204	0.09 ± .065	0.672
Degree of dehydration	Mild	17 (14.9)	14 (20.3)	0.556
	Moderate	89 (78.1)	49 (71)	
	Severe	8 (7)	6 (8.7)	

Data were presented as N (%) or Mean ± SD

Table 2 shows the distribution of DKA correction time, the timing of the initiation of dietary intake as well as the levels of HbA1c, insulin and C-peptide on the first day of hospitalization. The median of DKA correction time was 41.72 h and the timing of the initiation of dietary intake was 3.13 h. The median of HbA1c, insulin and C-peptide was 12.90%, 6.31 mU/L and 0.08 nmol/L, respectively.

**Table 2:** The distribution of timing of the initiation of dietary intake, HbA1c, insulin and C-peptide

	25 ^th^	50 ^th^	75 ^th^
DKA correction time (h)	23.28	41.72	59.70
Timing of the initiation of dietary intake(h)	1.78	3.13	16.23
HbA1c at admission (%)	11.20	12.90	14.00
Insulin at admission (mU/L)	1.84	6.31	11.65
C-peptide at admission (nmol/L)	0.04	0.08	0.12

HbA1c, insulin and C-peptide were collected on the first day of hospitalization

Table 3 showed the associations between the timing of the initiation of dietary intake with DKA correction. Specifically, with adjustment for potential confounders (child’s age, gender, weight, height and degree of dehydration), in multivariable regression models, for each hour delay in starting dietary intake, children’s DKA correction was prolonged by 0.5 (95% CI 1.05, 1.11, *P* < 0.001) hours. Accordingly, with each hour delay of the timing of the initiation of dietary intake, the adjusted odds of DKA over 48 h was increased by 8% (OR = 1.08, 95% CI: 1.05, 1.11, *P* < 0.001).

**Table 3:** Associations between timing of the initiation of dietary intake with DKA correction

	Model 1^a^β (95%CI)	*p*	Model 2^b^β (95%CI)	*p*
Timing of the initiation of dietary intake (h)	0.90 (0.67, 1.14)	**<0.001**	0.50 (0.30, 0.69)	**<0.001**
	Model 1^a^OR (95%CI)	*p*	Model 2^b^ OR (95%CI)	*p*
Timing of the initiation of dietary intake (h)	1.07 (1.04, 1.10)	**<0.001**	1.08 (1.05, 1.11)	**<0.001**

Model 1 was unadjusted model; Model 2 was adjusted for child’s age, gender, weight, height and degree of dehydration

**Fig. 1:**
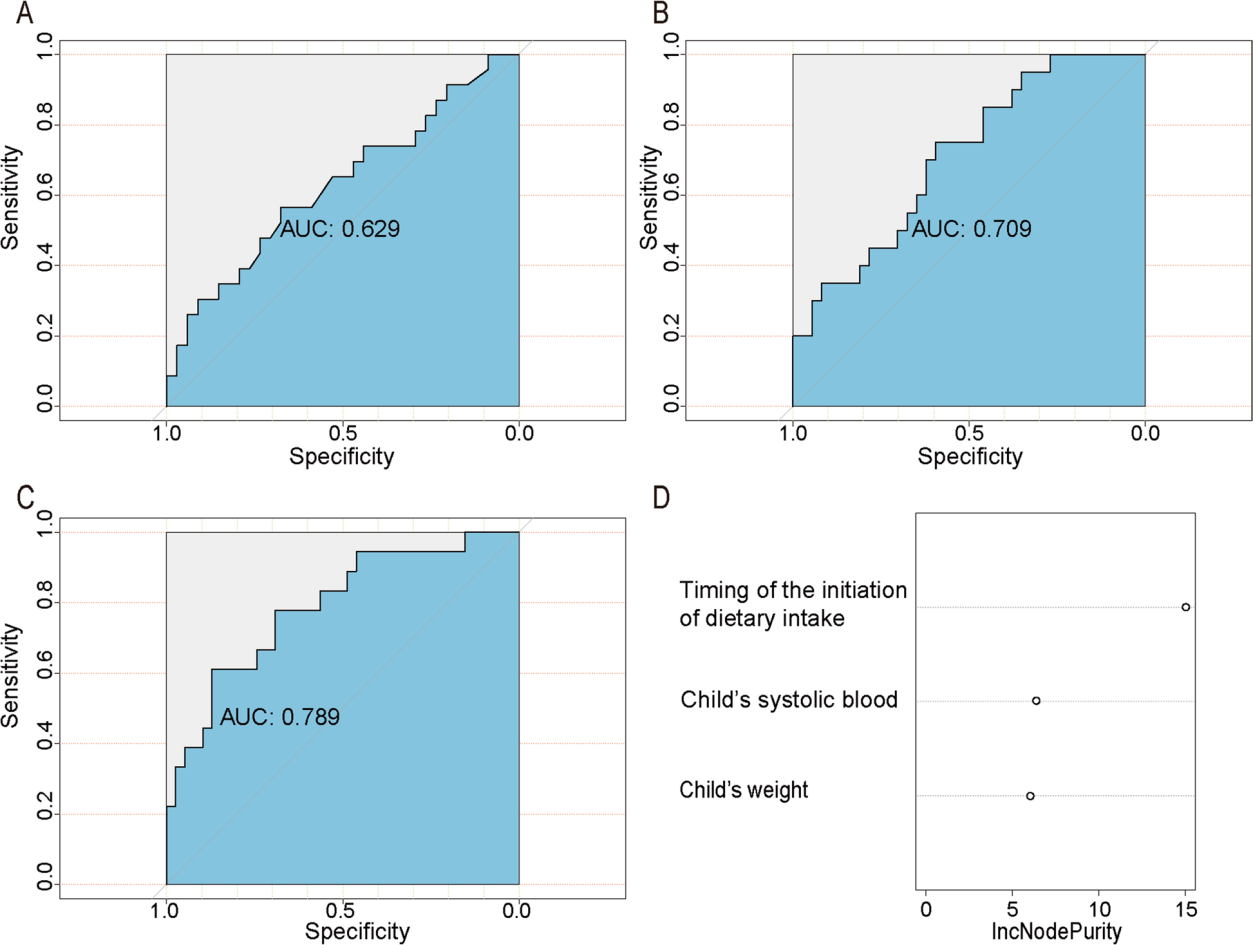
RF model for analyzing the effects of the timing of the initiation of dietary intake, child’s weight and systolic pressure on DKA correction. **A** RF model based on the timing of the initiation of dietary intake achieved the AUC of 0.629. **B** RF model based on the timing of the initiation of dietary intake and child’s weight achieved the AUC of 0.709. **C** RF model based on the timing of the initiation of dietary intake, child’s weight and systolic pressure achieved the highest AUC of 0.789. **D** The weight of the timing of the initiation of dietary intake, child’s weight and systolic pressure on DKA correction

RF model was used to analyze the independent effects of the timing of the initiation of dietary intake, as well as the timing of the initiation of dietary intake combined with positive individual basic features (child’s weight and systolic pressure) on DKA correction. According to the selected variables, different combinations of AUC were calculated. The model based on the timing of the initiation of dietary intake achieved the AUC of 0.629. The combined model with child’s weight achieved the AUC of 0.709, further, with both child’s weight and systolic pressure achieved the highest AUC of 0.789. The timing of the initiation of dietary intake contributed most, and followed by child’s systolic pressure (Fig. 1A-D).

Arterial PH, arterial HCO_3_^−^, blood glucose and blood ketone at multiple time points within 48 h after hospitalization were described as curves (Supplementary Fig. 1 A-D).

## Discussion

Precise therapy regimens of DKA correction in pediatric TID have always been the focus in endocrinology. Appropriate administration of intravenous rehydration and insulin therapy might correct DKA more quickly and reduce the incidence of cerebral edema and organ dysfunction; however, there were no data to support the timing of the initiation of dietary intake during rehydration and insulin therapy for DKA correction. Of note, in our real-world retrospective study involving 183 pediatric DKA cases, we documented that 62.3% of children corrected DKA at the end of the 48-h rehydration therapy with rehydration, insulin therapy and other supportive care methods strictly in accordance with guidelines for correcting pediatric DKA.

Previous prospective or retrospective studies explored the effects of different therapeutic factors or children’s own factors on DKA correction. Timing of dietary intake is one of the overlooked factors. Our retrospective study suggested an important role of the timing of the initiation of dietary intake during DKA correction, for each hour delay of the timing of the initiation of dietary intake, child’s DKA correction was prolonged by 0.5 h and the adjusted odds of DKA over 48 h was increased by 8%. Since the timing of the initiation of dietary intake can be intervened in clinical practice, therefore, our evidence emphasis the importance of early dietary intake during DKA correction.

Although there is no direct evidence to support our results, previous studies have explored the relationship between dietary factors and DKA. For example, one study involving adolescents with T1D suggested that eating disorders have more than triple the risk of DKA [16]. Another study investigated the association between different diet composition and risk of DKA in T1D [17]. Due to leptin’s acute insulin-independent effect to reverse DKA [18], and increased leptin secretion by food intake [19–21], we hypothesize that leptin may mediate the relationship between the timing of the initiation of dietary intake and DKA correction as an internal mechanism.

In addition to the timing of the initiation of dietary intake, we also found that in the group that corrected DKA for over 48 h, children were heavier and children had higher systolic blood pressure on admission. Hence, we used these three key variables (the timing of the initiation of dietary intake, child’s weight and systolic pressure) to establish the RF model. The model independently based on the timing of the initiation of dietary intake achieved the AUC of 0.629, and combined with child’s weight and systolic pressure achieved the highest AUC of 0.789, which suggested that under the premise procedures of pediatric DKA in guidelines, the combination of these three parameters is a good predictor of DKA correction. In addition, among these three variables, the timing of the initiation of dietary intake contributes most, from which the importance of early dietary intake is further emphasized.

The first advantage of our study was that we focused on the timing of the initiation of dietary intake in pediatric T1D for DKA, an understudied factor in special population. Given the increasing incidence of pediatric T1D for DKA, our finding may have implications for clinical treatment in the future. Second, the timing of the initiation of dietary intake is a controllable factor, and may provide a clue to correct DKA accurately. Third, the quality control of our study is strict. Rehydration, insulin therapy and other supportive care methods in our cases were followed the procedures of pediatric mild, moderate and severe DKA in guidelines. No cases developed cerebral edema during DKA correction. When analyzing the influence of the timing of the initiation of dietary intake on DKA correction, we adjusted the degree of dehydration as a confounder, and the model was stable.

There are also some limitations of this study. First, the results of our study are still limited by the relative low event rate in single center study and the lack of external validation. Second, due to our observational study, we cannot determine causality and cannot include unmeasured confounders. Third, our research focused on the timing of the initiation of dietary intake, and the amount, frequency, type and order of dietary intake during DKA correction are also factors that need to be considered in future studies.

## Conclusion

This research suggested that the timing of the initiation of dietary intake cannot be ignored in pediatric T1D for DKA, which highlights the need for pediatricians to pay attention to the timing of the initiation of dietary intake, a controllable factor that might contribute to reducing time to correction of DKA.

## Supplementary Information

**Additional file 1: Supplementary Figure 1**. Arterial PH, arterial HCO3^−^, blood glucose and blood ketone at multiple time points within 48 h after hospitalization. A. Curve of four arterial PH tests. B. Curve of eight blood glucose tests. C. Curve of four arterial HCO3^−^ tests. D. Curve of nine blood ketone tests.

## Data Availability

The datasets used and/or analysed during the current study available from the corresponding author on reasonable request.
