# Supplementary material for: Effects of the timing of the initiation of dietary intake on pediatric type 1 diabetes for diabetic ketoacidosis
Source: BMC Pediatr. 2022 Apr 13;22:206. doi: 10.1186/s12887-022-03243-z (PMC9008930; doi:10.1186/s12887-022-03243-z)
Supplement: Supplementary file 1 — Additional file 1: Supplementary Figure 1. Arterial PH, arterial HCO3−, blood glucose and blood ketone at multiple time points within 48 h after hospitalization. A. Curve of four arterial PH tests. B. Curve of eight blood glucose tests. C. Curve of four arterial HCO3− tests. D. Curve of nine blood ketone tests. [file 12887_2022_3243_MOESM1_ESM.docx]

**
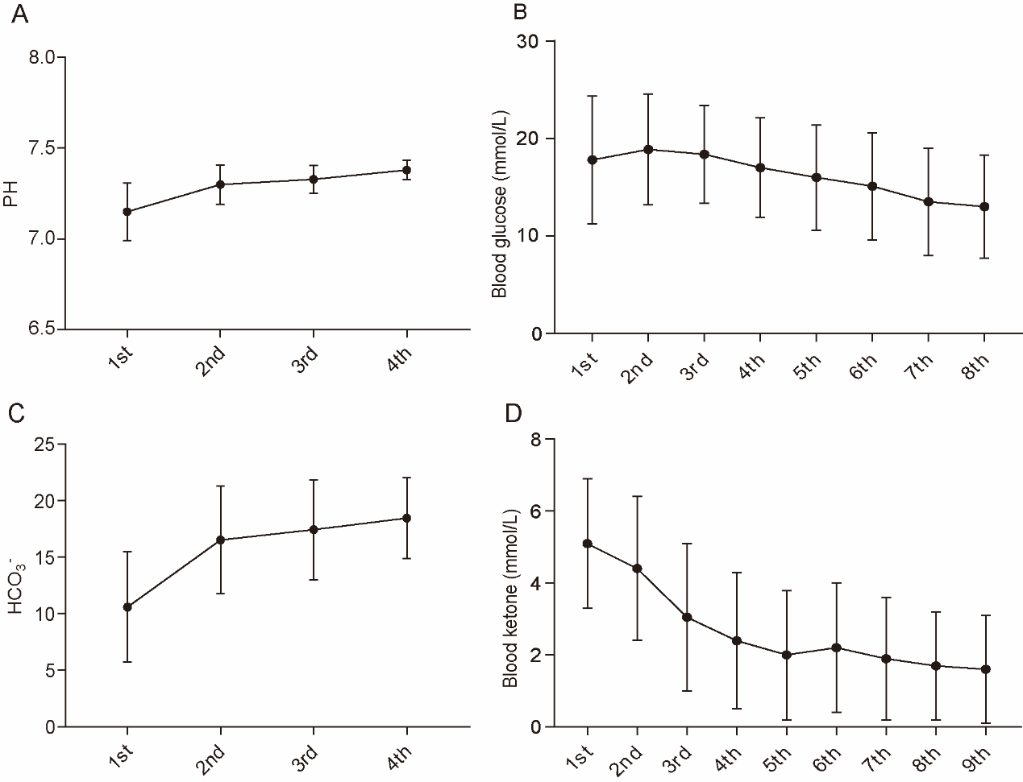
**

**Supplementary Figure** **1** Arterial PH, arterial HCO3^-^, blood glucose and blood ketone at multiple time points within 48 hours after hospitalization. A. Curve of four arterial PH tests. B. Curve of eight blood glucose tests. C. Curve of four arterial HCO3^-^ tests. D. Curve of nine blood ketone tests.
